# Bioarchaeological and paleogenomic profiling of the unusual Neolithic burial from Grotta di Pietra Sant’Angelo (Calabria, Italy)

**DOI:** 10.1038/s41598-023-39250-y

**Published:** 2023-07-24

**Authors:** Francesco Fontani, Rosa Boano, Alessandra Cinti, Beatrice Demarchi, Sarah Sandron, Simone Rampelli, Marco Candela, Mirko Traversari, Adriana Latorre, Rocco Iacovera, Paolo Abondio, Stefania Sarno, Meaghan Mackie, Matthew Collins, Anita Radini, Chantal Milani, Enrico Petrella, Emanuela Giampalma, Antonella Minelli, Felice Larocca, Elisabetta Cilli, Donata Luiselli

**Affiliations:** 1 Department of Cultural Heritage, University of Bologna, Via Degli Ariani 1, 48121 Ravenna, Italy; 2 Department of Life Sciences and Systems Biology, University of Turin, Via Accademia Albertina 13, 10123 Torino, Italy; 3 Department of Pharmacy and Biotechnology, University of Bologna, Via Belmeloro 6, 40126 Bologna, Italy; 4 Department of Biological, Geological and Environmental Sciences, University of Bologna, Via Selmi 3, 40126 Bologna, Italy; 5 Faculty of Health and Medical Sciences, The Novo Nordisk Foundation Center for Protein Research, University of Copenhagen, Blegdamsvej 3B, 2200 København, Denmark; 6 Faculty of Health and Medical Sciences, The Globe Institute, University of Copenhagen, Øster Farimagsgade 5, 1353 København, Denmark; 7 School of Archeology, University College Dublin, Belfield, Dublin 4, Ireland; 8 McDonald Institute for Archaeological Research, University of Cambridge, Downing Street, Cambridge, CB2 3ER UK; 9 SIOF – Italian Society of Forensic Odontology, Strada Degli Schiocchi 12, 41124 Modena, Italy; 10 Radiology Unit, Morgagni-Pierantoni Hospital, AUSL Romagna, Via Carlo Forlanini 34, 47121 Forlì, Italy; 11 Department of Humanities, Education and Social Sciences, University of Molise, Via Francesco De Sanctis, 86100 Campobasso, Italy; 12 Speleo-Archaeological Research Group, University of Bari, Piazza Umberto I 1, 70121 Bari, Italy; 13 Speleo-Archaeological Research Centre “Enzo dei Medici”, Via Lucania 3, 87070 Roseto Capo Spulico (CS), Italy

**Keywords:** Proteomic analysis, Anthropology, Archaeology, Genome, Haplotypes, DNA sequencing, Next-generation sequencing, Imaging techniques

## Abstract

The Neolithic burial of Grotta di Pietra Sant’Angelo (CS) represents a unique archaeological finding for the prehistory of Southern Italy. The unusual placement of the inhumation at a rather high altitude and far from inhabited areas, the lack of funerary equipment and the prone deposition of the body find limited similarities in coeval Italian sites. These elements have prompted wider questions on mortuary customs during the prehistory of Southern Italy. This atypical case requires an interdisciplinary approach aimed to build an integrated bioarchaeological profile of the individual. The paleopathological investigation of the skeletal remains revealed the presence of numerous markers that could be associated with craft activities, suggesting possible interpretations of the individual’s lifestyle. CT analyses, carried out on the maxillary bones, showed the presence of a peculiar type of dental wear, but also a good density of the bone matrix. Biomolecular and micromorphological analyses of dental calculus highlight the presence of a rich Neolithic-like oral microbiome, the composition of which is consistent with the presence pathologies. Finally, paleogenomic data obtained from the individual were compared with ancient and modern Mediterranean populations, including unpublished high-resolution genome-wide data for 20 modern inhabitants of the nearby village of San Lorenzo Bellizzi, which provided interesting insights into the biodemographic landscape of the Neolithic in Southern Italy.

## Introduction

At the end of the sixth millennium BCE, Neolithic lifeways are well established in Southern Italy (SI)^1^. Pottery appears in the archaeological record of the Early and Middle Neolithic phases in Apulia, Basilicata and Calabria; the hunting-gathering economic system starts to be replaced by the spread of farming activities, even though a complete shift to an agriculture-based subsistence strategy does not occur before the Bronze Age^2^. Radical changes can be seen in the settlement strategies during the Early and Middle Neolithic phases in SI, mostly based on the occupation of coastal and sub-coastal areas, such as Favella di Corigliano (CS), representing one of the most ancient farming villages in Italy, and one of the first pottery-industry in Central-Western Europe^3^. By contrast, evidence of human presence in the inner territories of SI are sporadic until the advanced phases of the Neolithic. In such context, the archaeological site of Grotta di Pietra Sant’Angelo represents a unicum for the human exploitation of SI during the Neolithic.

**Figure 1:**
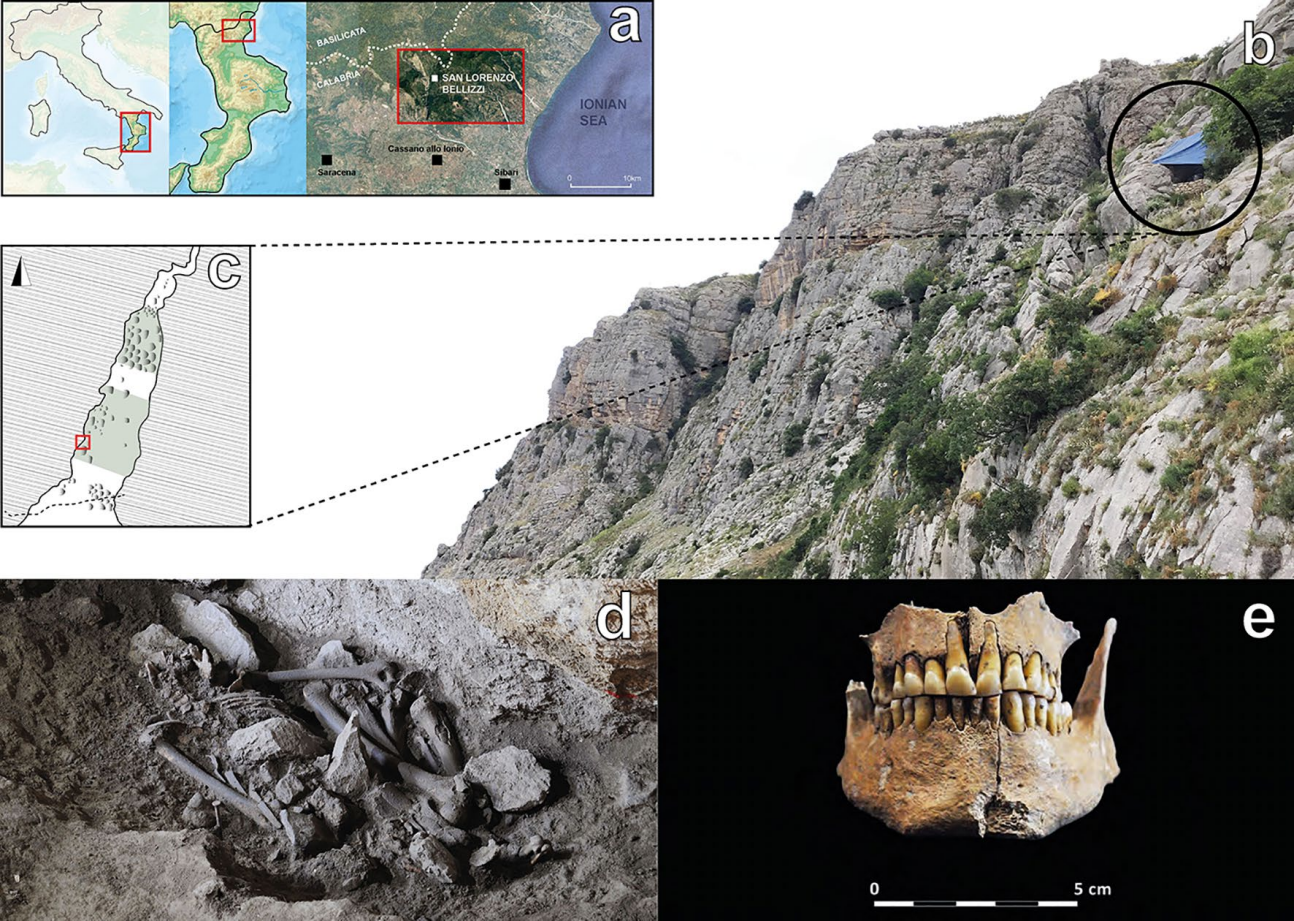
(**a**) Geographical location of San Lorenzo Bellizzi. Maps were obtained from the National Geologic Map Database project (https://ngmdb.usgs.gov/topoview/) and adjusted using Adobe Illustrator 2022 v3.0 (available at https://www.adobe.com/products/illustrator/); (**b**) Pietra Sant’Angelo massif and the entrance of the homonymous cave (black circle); (**c**) planimetry of the cave and location of the burial (red square); extent of the excavations is highlighted in green; (**d**) the skeletal remains of the individual as found inside the cave; (**e**) the intact jaws of the individual; photographs taken by Felice Larocca (**b**, **d**) and Alessandra Cinti (**e**).

Pietra Sant’Angelo is an extended limestone massif located on the Calabrian side of the Pollino National Park (Fig. 1a), in the municipality of San Lorenzo Bellizzi (Cosenza). The massif is extremely rich in cavities, with at least 21 vertical and horizontal caves surveyed in the area. Among them, Grotta di Pietra Sant’Angelo preserves the most ancient evidence of human activity in Northern Calabria^4^. The location of the cavity (Fig. 1b) at a rather high altitude (> 1000 m asl) and not easily visible from the valley, has allowed it to survive post-depositional disturbances. The stratigraphic reconstruction of the archaeological deposit testifies a consistent use of the small cavity (less than 20 m in length) throughout the Neolithic period, as suggested by the presence of trichrome, bichrome and impressed ware, while scarce and sporadic evidence of a more recent frequentation has been attested.

The archaeological excavations conducted since 2017 revealed the presence of a single burial located a few meters from the entrance of the cavity (Fig. 1c). The skeletal remains (Fig. 1d) were found inside an ovular pit directly carved in the ground: the buried individual (SLB henceforth) was found prone, E–W oriented, face turned to the earth and in an extremely contracted position; medium-sized stones covered the skull and the right side of the body, with smaller stones lying above the pelvis, trunk, and limbs. Despite a consistent anthropogenic presence in the cave, marked by evidence of pottery fragments associated with the Diana and Serra d’Alto *facies*^5^, the cave did not yield any further signs of funerary activity. The lack of funerary equipment, along with the use of a simple pit inside a cave, led to a Neolithic contextualization of the burial. This was later confirmed by radiocarbon analysis, which placed SLB within the Middle Neolithic (6092 ± 45 BP; 5207–5048 cal BCE, 19,1%/5084–4899 cal BCE, 74,8%/4864–4853 cal BCE, 1,5%)^6^. However, the individual from Grotta di Pietra Sant’Angelo represents an interesting exception in the funerary landscape of the Italian Neolithic: single inhumations, during the earliest and the more advanced phases of Neolithic in SI, are generally found within the domestic area, with the body lying on one side, mostly buried in pits directly carved in the ground or inside silos and niches^7–9^. Evidence of mortuary depositions far from inhabited areas can be found throughout the Neolithic in proper Apulian funerary contexts, such as necropolis^10^ and burial cavities^11,12^. However, considering the location of the cave and the problems that arise in accessing it, due to the presence of steep vertical crags, this burial constitutes a unique archaeological marker of human Neolithic presence in the mountains of SI. Moreover, the unusual placement of the burial inside the cave, located only a few meters from the entrance and partially exposed to natural light, raises questions on the funerary habits of Neolithic humans in SI, and more specifically on the life history of the individual. We wondered if the genetic ancestry, the state of health, occupation or social identity could somehow be connected to the unconventional burial. A well-established methodological approach combining biomolecular archaeology, physical anthropology and archaeothanatology^13–16^ was able to provide answers to some of these questions, and helped us reconstruct an almost complete bioarchaeological profile of the individual. Anthropological and paleopathological investigations of the remains have been supplemented with computerized tomography scanning of the mandible and the maxilla (Fig. 1e). The presence of dental calculus has allowed the characterization of the microdebris trapped in the mineral matrix, as well as the recovery of interesting information on the composition of the oral microbiome and the potential state of disease thanks to the combination of metagenomics and paleoproteomics analyses. Finally, a complete mitochondrial genome and the first whole genome data from prehistoric Calabria have been produced through shotgun sequencing. In fact, while ancient DNA approaches have been applied to investigate the Neolithic transition in Northern^17–19^, Central^20^, and insular Italy^21–23^, few ancient DNA data have been produced yet for prehistoric SI^24–26^ and very little is known about the paleogenomic landscape of Calabria^27^. Thus, genomic data obtained here were investigated in the context of modern and ancient variability of Mediterranean populations. To improve our understanding of population dynamics, samples of 20 modern autochthonous individuals from the village of San Lorenzo Bellizzi were collected and genotyped for ~ 720,000 genome-wide single nucleotide polymorphisms (SNPs).

## Results

### Anthropological investigation

**Figure 2:**
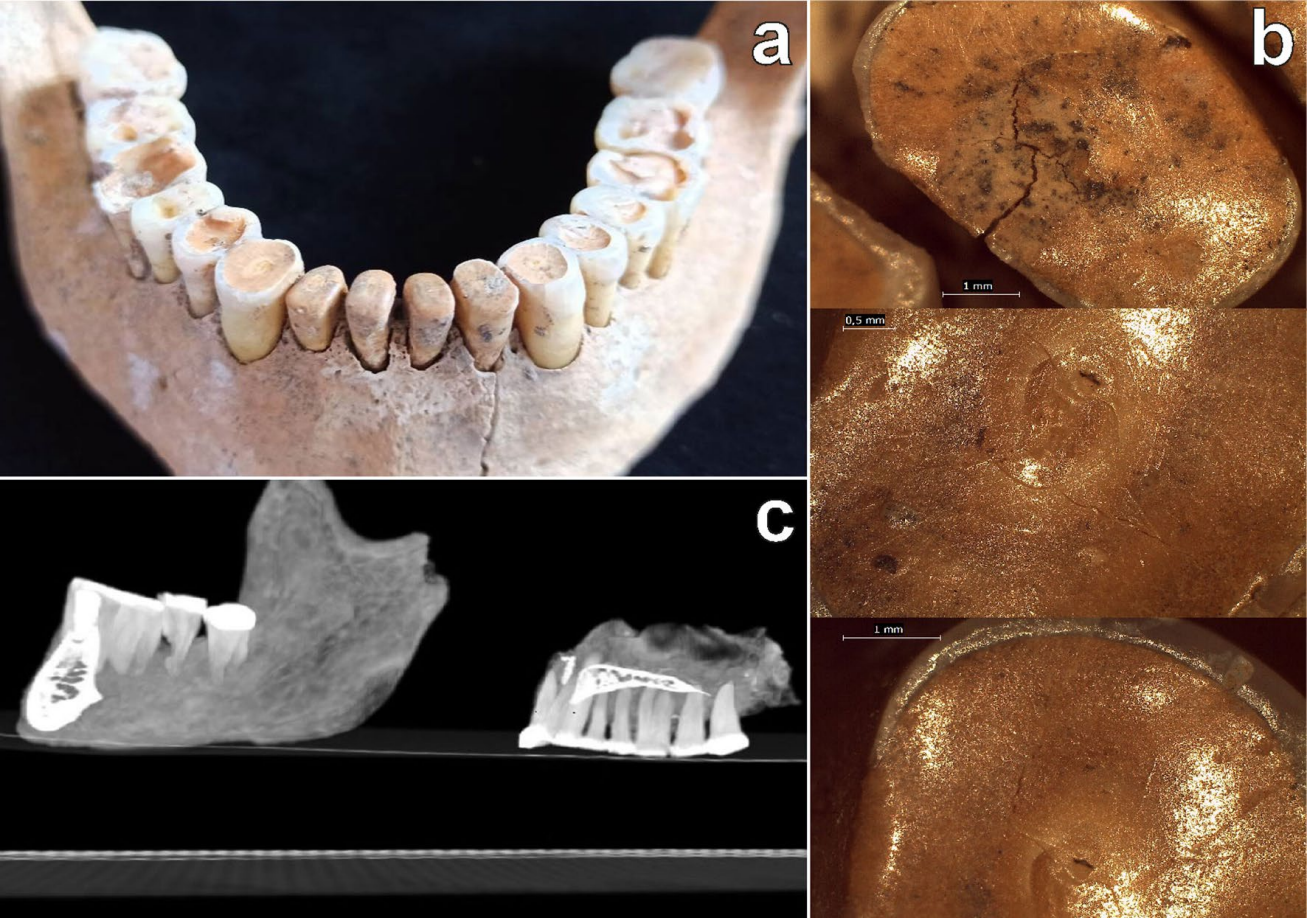
(**a**) Mandibular portion of the jaws with visible extreme forms of wear patterns; (**b**) stereomicroscopic images of the occlusal surfaces of lower incisors and canines, with clear evidence of streaks (bottom and central) and fractures (top); (**c**) CT images of the jaws.

The skeletal remains of the individual were found in a very contracted position, with the torso prone, the face resting on the bottom of the pit, with elbows and knees bent; both forearms were placed under the body, with the lower limbs reassembled on the right side and arranged with the legs hyper-flexed on the thighs. The mandible and the maxilla were found in full occlusion. The primary character of the deposition has been defined by the correct anatomical arrangement of the skeletal elements and by the persistence of labile connections; the absence of disjunction of the vertebral spaces, the maintenance of the volume of the rib cage, and the anatomical connection of the bones of the hands and feet suggest that the decomposition occurred in a filled pit^28^. The individual, an adult male ± 30 years old, about 164 cm ± 3 cm tall, showed extreme wear patterns on the teeth (Fig. 2a), involving the complete loss of crown height in the lower incisors. Dental enamel is almost absent from the occlusal surface, leaving a nearly continuous dentinal surface surrounded by a partial rim of enamel, and causing a remarkable reduction in tooth size.

Numerous streaks were visible by stereomicroscopy in all directions on the occlusal surfaces of lower incisors and canines (Fig. 2b), as well as crown fractures and signs of chipping on the anterior mandibular and maxillary teeth. A more pronounced oblique wear on the lingual surface of the maxillary teeth and on the buccal surface of the mandibular teeth (friction) is present, along with signs of wear on the tooth surface (abrasion), particularly evident on the mandibular and maxillary first molars; chipping and fractures of the crowns on the posterior teeth (molars) are also visible. Diffuse recession of the alveolar bone and exposure of the dental roots are present as well as an *intra-vitam* loss of the second maxillary right premolar, probably following an abscess within the alveolar bone. Small amount of calculus on the teeth surfaces were also identified.

CT-investigation of the jaws (Fig. 2c) reported a good density of the bone matrix, consistent with the age of the individual; a generalized bone absorption is visible at the alveolar level, indicating the presence of a periodontal disease that could have led to the loss of the maxillary second premolar. The relationships between the two jaw bones show a forward sliding of the lower jaw (class III) in head-to-head closure of the incisors. It allows us to estimate an original position of the lower jaw (in class I) that changed over time due to the lack of occlusal mandibular and maxillary stability.

Regarding the postcranial skeleton, abnormal bone formation or destruction patterns were observed at the level of the joints. Moderate marginal osteophytes have been detected on the right and left upper extremity (proximal epiphysis of the right ulna and distal epiphysis of the right and left radius) and on the pelvic girdle (left acetabulum). Depressed surfaces with smooth edges were observed on the right distal surface of humeral trochlea (10 mm long), on posterior surface of the left and right tarsal navicular bones (about 3 mm long) and on the first proximal phalanx of the right toe (concal proximal facet, 10 mm long). Such patterns are typical of osteochondritis dissecans, a pathological condition of the subchondral bone supporting cartilage of synovial joints, consisting in the partial or complete detachment of articular cartilage from subchondral bone^29^. Patterns of abnormal bone formation and destruction were identified at the shafts. Non-specific periostitis on the midshafts of tibiae and fibulae is visible, and moderate abnormal bone formations in the form of enthesopathies are present, affecting the inferior surface of both clavicles at the attachment of the ligament of the conoid process and the sites of flexor tendons on palmar surface of proximal hand phalanges. Severe oval grooves are evident along the inferior surface of the right clavicle at the attachment of the costoclavicular ligament (rhomboid fossa); on the left clavicle the lesion appears as moderate depression (enthesopathies), while on the upper humeral shaft, moderate depression at the attachment of the pectoralis major muscle is present. Abnormal bone shapes have also been detected, involving a probable greenstick fracture of the right clavicle and backward inclination of the tibial plateau.

Finally, a lateral extension of the articular surface of the right glenoid cavity occurs on the insertion of inferior glenoid labrum; no ossification of ligaments and tendon attachment are present as well as no osteophytes, indicating a possible instability or flexibility of the shoulder joint. Squatting facets are present on the anterior surface of the trochlea of talus and on the anterior margin of the lower end of the tibial extension, indicating a habitual contact between the two anatomical elements^29^.

### Dental calculus analysis

The microdebris analysis of dental calculus revealed the presence of a variety of poorly diagnostic microremains (Supplementary Information). Most of these were of plant origin and consisted of starch granules (Supplementary Fig. 2A), plant tissues (Supplementary Fig. 2B), a trichome (plant hair, Supplementary Fig. 2C), but in most cases they could not be assigned even to Family level. Two of the plant remains retrieved were consistent with fibers (Supplementary Fig. 2D,E), but they were of low diagnostic nature, too. Dark brown particles (Supplementary Fig. 2G) might be evidence of micro-charcoal, but they also lacked secure distinctive features, therefore they were described as ‘burnt debris’—to acknowledge their uncertain nature. There was also mineral grit (Supplementary Fig. 2F), but optical microscopy alone does not allow the identification of such remains, and it will be subject of future analysis. Despite the poor diagnostic nature of the remains, they suggest the consumption of starchy food and leafy crops. The presence of remains such as fibers and mineral grit, that may have contributed to the dental wear of the individual, could also be associated with occupational activity. However, the very limited quantity of potential textile fibers and their insecure identification make it difficult to provide a clear interpretation. In fact, fibers can also be found in calculus sample where no dental wear patterns are reported^30^, and thus extreme caution must be taken in the interpretation of these remains.

**Figure 3:**
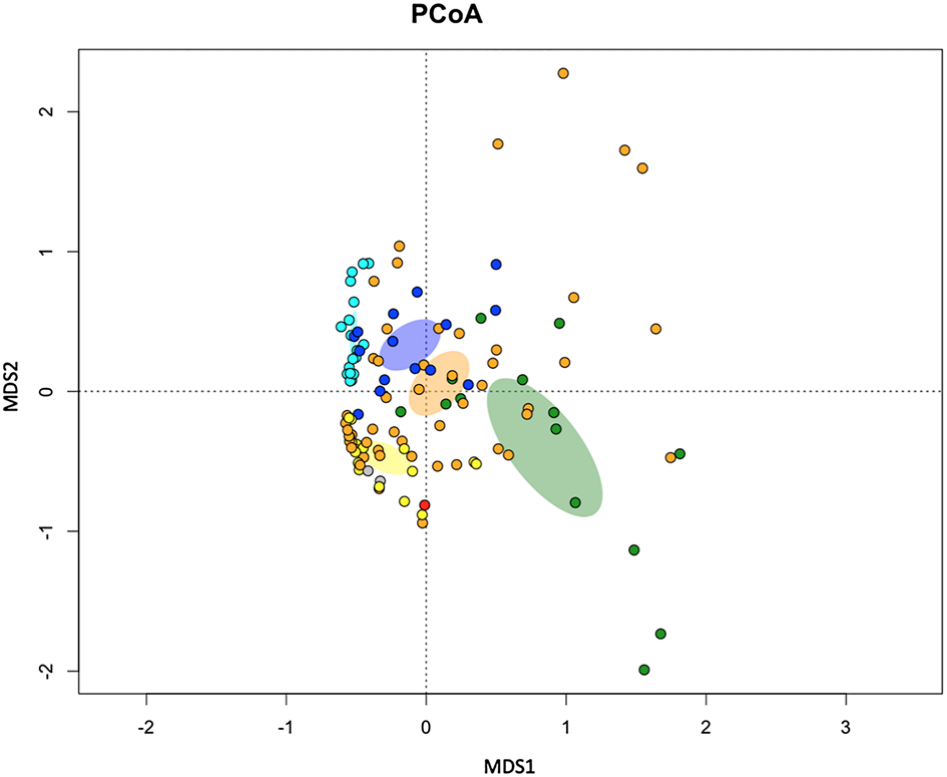
Principal Coordinates Analysis (PCoA) of the ancient oral microbiome content for SLB (red) and 114 samples from Neanderthal (green), pre-agricultural (orange), Neolithic (yellow), pre-antibiotic period (blue) and modern-day humans (light blue). Ellipses indicate the 95% confidence areas based on the standard error of the means of distances among samples of the same group.

Paleogenomic analysis was performed on dental calculus to define the ancient oral microbial community of SLB. Single-stranded genomic libraries were built from ancient DNA extracts of dental calculus and sequenced on Illumina HiSeq X platform, producing 7,695,982 paired-end reads. We applied HOPS^31^, a tool specifically conceived for the taxonomic characterization of short-read shotgun metagenomic data. Several host-associated bacteria have been identified in the dental calculus, among which different members of the genera *Treponema, Prevotella, Streptococcus and Methanobrevibacter* are present (a list with the 50 most abundant microbial species identified in the dental calculus is reported in Supplementary Table S1)*.* As expected for dental calculi samples, members of bacterial genera associated with both human host and environment (e.g., *Actinomyces* spp.*, Streptomyces* sp. *and Pseudomonas* spp.) were also detected. The ancient origin was validated by evaluating the number of reads for each specific taxon, the post mortem degradation score (PMDS), the negative difference proportion score (−Δ %) and the presence of pattern of deamination C-T. Taxa with more than 300 assigned reads, more than 50 reads with PMDS > 1, − Δ % > 0.9 and showing patterns of C-T transition at 5′ were considered to be of ancient origin (Supplementary Table S1) A large number of reads assigned to previously identified human oral taxa sustained deamination and substitution profiles that are consistent with ancient DNA (e.g., *Treponema denticola*, *Actinomyces dentalis*, *Streptococcus gordonii*, *Methanobrevibacter oralis*, *Porphyromonas gengivalis*, *Prevotella intermedia*). Of the species retrieved, *Treponema denticola*, *Tannerella forsythia* and *Porphyromonas gingivalis* constitute the most pathogenic aggregate of oral bacteria, namely the “red complex”, recognized to be strongly associated with periodontal disease^32,33^. The occurrence of these three species in terms of number of reads (Table S1) is in line with the expected abundance, as highlighted in other studies^34^. The oral metagenomes detected from dental calculus were compared in Principal Coordinates Analysis (Fig. 3) with available ancient oral microbiomes data of Neanderthal (n = 14, green), pre-agricultural (n = 51, orange), Neolithic (n = 16, yellow), pre-antibiotics (n = 15, blue) and modern (n = 18, light blue) origin^35,36^. Notably, 16 of these metagenomes belong to ancient Italian individuals geographically and temporally matching with our sample. The oral microbiome of SLB (red dot) lies in proximity to samples of Neolithic/pre-agricultural origin, indicating a major sharing of taxa with microbiomes of this period as expected by the age of the sample. To further investigate the metagenomic component of the individual, ancient DNA sequences generated from tooth dentin of SLB (see section “Ancient human DNA”) were also screened for the most common bacterial pathogens, as suggested by the literature^37,38^. According to our findings, no DNA traces of potential pathogens were found (Supplementary Table S2). It must be noted however that pathogen DNA may not have been preserved through time on the dentin due to the conformation and thickness of the different bacterial cell walls^39^ or because some pathogens do not enter the bloodstream and are most likely undetectable^40^. Moreover, our analysis is completely blinded to viruses, especially RNA viruses, whose genetic material is hardly preserved in ancient samples, and few attempts to sequence ancient transcriptomes have been published so far^41^.

Paleoproteomics of a small dental calculus sample yielded 1309 unique protein hits (Supplementary Dataset). Among these, 27 belong to the host (Supplementary Table S3) and most (20) are associated with the innate immune system (Supplementary Fig. S3). Others are related to hemostasis, the metabolism of proteins and the digestion of dietary carbonate (amylases). Most of the identified proteins were of bacterial origin (1271). The analysis of the taxonomic composition of the ancient microbiome is complicated by the fact that the same peptide sequence may be found in several taxa, leading to multiple hits. Nonetheless, considering the taxa that yielded the highest number of protein hits, *Arachnia* spp., *Actinomyces* spp., *Streptococcus* spp., *Fusobacterium* spp., *Corynebacterium* spp., *Olsenella* spp. and *Ottowia* spp. are the most represented. All these taxa are components of the healthy core oral microbiome (eHOMD https://www.homd.org, accessed 11/01/2023). The presence of *Treponema* spp. and *Tannerella* spp., present among the “red complex” bacteria, is confirmed also from the proteome. Other bacteria of potential interest include *Aggregatibacter* spp*.*, *Cardiobacterium* spp*.* and *Eikenella corrodens*, regular components of a healthy microbiome, but which can, in rare cases, be the cause of infective endocarditis^42^.

### Ancient human DNA

**Figure 4:**
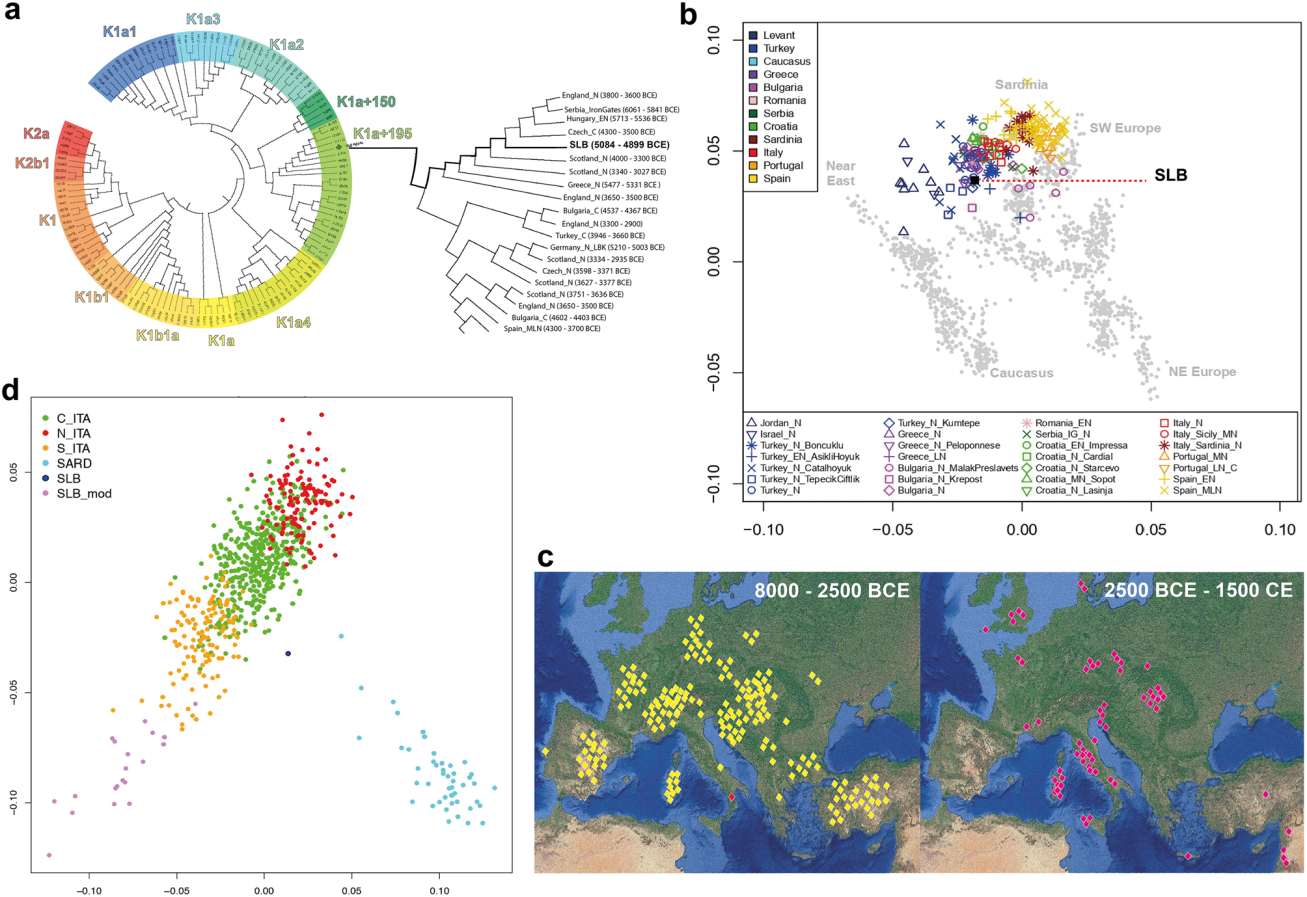
(**a**) Phylogenetic reconstruction of ancient K-related mitochondrial lineages shows SLB falling into the variability of K1a + 195 haplogroup (olive green); (**b**) PCA projection of SLB individual into the variability of ancient Italian and Mediterranean Neolithics; (**c**) the distribution of G2a-related Y-chromosome lineages in ancient Europe between 8000 and 2500 BCE (left) and 2500 BCE—1500 CE (right). SLB is shown in a red square. Genomic data taken from the David Reich Lab Allen Ancient DNA Resources v54.1. Maps were obtained from the National Geologic Map Database project (https://ngmdb.usgs.gov/topoview/) and adjusted using Adobe Illustrator 2022 v3.0 (available at https://www.adobe.com/products/illustrator/); (**d**) when projected into the variability of modern Italians, SLB (blue dot) clusters in close proximity to the modern southern Italians (yellow dots) but does not overlap with them.

Ancient DNA was extracted from one molar of SLB to test for endogenous DNA persistence and provide a genetic profile of the individual. The extracts were converted into single-stranded genomic libraries and sequenced for a total of 531 million reads on Illumina HiSeq-X Ten Platform. The endogenous DNA retrieved reported a genome-wide coverage of 0.24X, with > 290,000 SNPs overlapping the 1240 K—Human Origins SNP Array v54.1 (https://reich.hms.harvard.edu/allen-ancient-dna-resource-aadr-downloadable-genotypes-present-day-and-ancient-dna-data). Deamination patterns among the sequences were consistent with the age of the sample (Supplementary Fig. S4), and the presence of modern contamination was excluded both at the X-chromosome (6%) and mitochondrial level (2%) (Supplementary Table S4). A 39-fold complete mitochondrial genome was reconstructed from the individual, reporting 33 unique variants when compared to the rCRS. The mitochondrial haplogroup has been assigned with 99% accuracy to K1a + 195 haplogroup. The Y-chromosome haplogroup was identified as a G2a2a1 by comparing high quality reads of SLB to an informative list of Y-chromosome SNPs (https://isogg.org/tree/). Both the X/autosomes coverage ratio and Y/sex chromosomes coverage ratio suggest the individual to be male, confirming the outcome of the anthropological investigation. A phylogenetic tree (Fig. 4a) was reconstructed to investigate the clustering of the individual in the landscape of prehistoric K-related mitochondrial lineages, and SLB clusters in proximity with other K1a + 195, in particular with one individual from the Neolithic settlement of Makotrasy (I14173, Baalberge culture, Czech Republic) dated 4300–3500 BCE. Interestingly, the MJN performed for mitochondrial variability shows SLB being few nodes far from another K1-related prehistoric individual from Italy, the Iceman^43^, whose rare mitochondrial haplogroup has been identified only within the Alps area (Supplementary Fig. S5).

Under a diachronic and geographical population perspective, we compared our sample with ancient and modern populations using principal component analysis. PCA projection on a dataset of ancient samples shows how SLB falls into the variability of the Italian and Mediterranean Neolithic, near Peloponnesian and Anatolian Neolithic individuals (Fig. 4b).

The SLB individual was then projected on a dataset of 737 modern Italians (Fig. 4d), representative of the genetic variability of Italy (see “Material and methods” section), merged with unpublished genomic data obtained from 20 modern inhabitants of the nearby municipality of San Lorenzo Bellizzi. The geographic clustering of the samples reveals a clear latitudinal gradient along the peninsula, where the Sardinian cluster is clearly separated from the rest. The modern inhabitants of San Lorenzo Bellizzi cluster together, in close proximity to the Southern Italian control group, but do not overlap with it; instead, they appear separate from the rest of the modern samples, indicating a possible isolation and a subsequent genetic drift effect. Despite being geographically close, the Neolithic individual SLB does not fall within the variability of modern groups, within that of the modern inhabitants of San Lorenzo Bellizzi, nor within the genetic variability of the South.

## Discussion

Grotta di Pietra Sant’Angelo is located at a substantial altitude, on a vertical crag that is extremely difficult to reach. Despite the many cavities that have been surveyed in the area of the rock massif, this is the only one where the presence of Neolithic funerary activities has been identified thus far. The typological characteristics of the burial found inside the cave designate it as a remarkable finding for the prehistory of Southern Italy. Similar types of deposition, with a single individual buried in a prone and huddled position, with the face turned towards the ground, can be found in Palata di Canosa^44^, Pulo di Molfetta^45^ and Titolo di Bari-Palese^9^, three Apulian sites dated to the Ancient and Middle Neolithic. It must be noted however that these burials are located in typical funerary contexts, within or in proximity of the villages. If one considers (1) the more general typological evidence of burying the deceased in a huddled position inside the pit, (2) the widespread presence of covering stones, and (3) the total absence of funerary equipment, more similarities can be found with the infant burial of Grotta di San Michele in Saracena^46^ and in the funerary area of the Neolithic village of Favella^3^. However, the social meanings that underlie the heterodox funerary behaviour expressed in Grotta di Pietra Sant’Angelo are difficult to be addressed. When compared to other similar archaeological contexts in Northern Ionian Calabria, such as the nearby caves of Cassano allo Ionio^47^, the absence of further human remains suggests that Grotta di Pietra Sant’Angelo had a marginal role as a burial site during the Middle Neolithic. Moreover, the extreme contraction of the body inside the pit proposes two interpretative scenarios: a critical frailty of the subject, which could have allowed the lower limbs to be easily flexed; the possible presence of ropes that kept the limbs together. The arrangement of the body with stones covering its entire extent could be associated with the practical aim of locking the body in the lying position. Furthermore, the completely unnatural position of the upper limbs and the hands could also indicate that the body has been arranged in such a position during the burial, either at a moment close to death, or in a later phase of at least 24–36 hours, i.e. the necessary time whereupon rigor mortis ceases^48^. Despite the lack of anthropological elements that could be directly related to the cause of death, several skeletal features identified through macroscopic investigation allow us to make some considerations on the lifestyle of the individual. The analysis of the post-cranial skeleton provides information related to acute or repeated moderate microtrauma on the shoulders (suspected greenstick fracture, enthesopathies), upper limbs (osteophytes, osteochondritis dissecans, enthesopathies), pelvic girdle and lower limbs (osteophytes, periostitis, osteochondritis dissecans). These alterations might be related to a general biomechanical stress on bones and joints that could also generate peripheral aspecific bone inflammation in the lower limbs^29,49^. In addition, bilateral squatting facets of the tibia and talus, and the backward inclination of the tibial plateau have been taken as evidence of hyper-flexion at the hip and knee joints and hyper-dorsiflexion at the ankle and subtalar joints; these joint markers are related to habitual use of the squatting position during life as well as prolonged standing and walking on hard/impervious surfaces^50–52^ and/or to genetic traits^53^. Overall, stress-induced skeletal remodeling involving different bones in a moderate way, suggests that a variety of repeated physical activities could have played a role in the lifestyle of the individual. The analysis of the dental wear provides evidence for the use of the mouth for non-alimentary purposes, as a non-specific tool or "third hand". The extreme forms of wear patterns of the lower incisors and the presence of micro-grooves on the occlusal surfaces have been taken as evidence of extra-masticatory activities and the reduction in tooth size has been described in literature as the results of the combined effects of occlusal and interproximal wear^54,55^. Dental wear patterns of masticatory type attributable to friction and abrasion have been observed in posterior teeth, even if the asymmetrical wear (more prominent on the right side) might be a diagnostic element related to activities such as the preparation of food or the processing of hard or resistant materials. Furthermore, the microdebris identified through morphological investigation confirms the utilization of the mouth for non-alimentary purposes. No diagnostic elements relating to the diet were found, but the presence of magenta minerals and fibers could be associated with the use of the mouth as a third hand during the production of craft objects. While it is difficult to link these fibers securely to a craft activity, the presence of marked dental wear on the teeth of SLB and the presence of fibers could be an indication of some fiber-based craft. Such evidence has been attested in other coeval funerary contexts in SI, such as Serra Cicora in Apulia, and associated to an employment of the mouth as a working tool^10^.

Among the dental pathologies detected, chipping and fractures of the tooth crowns have been taken as evidence of chewing of hard particles^56,57^. Finally, diffuse recession of the alveolar bone and exposure of the dental roots have been related to inflammation of soft tissues (periodontal disease, gingivitis)^58^. This paleopathological evidence is concordant with the biomolecular data obtained from dental calculus.

Paleoproteomics analyses of the calculus showed the presence of a rich oral microbiome, dominated by bacterial taxa commonly found in plaque and oral cavity. The authenticity of non-human peptide sequences, which could indicate the presence of dietary proteins (e.g., *Bos taurus* collagen), cannot be firmly established and therefore no further interpretation is attempted. The metaproteome data are consistent with the metagenomic analyses, showing the presence of *Actynomyces* and *Streptococcus* as well as of *Treponema* and *Tannerella* spp*.,* the latter being typical of periodontal disease. In particular, metagenomic analysis highlighted the persistence of the Gram-negative bacteria *Treponema denticola*, *Tannerella forsythia* and *Porphyromonas gingivalis*, which are part of the so-called “red complex” bacteria, known to be strongly associated with periodontal disease^32,33^, but also to a higher risk of developing esophageal cancer^59,60^, diabetes mellitus^61,62^, and are proposed as a risk factor for other several syndromes^63,64^. Among the bacteria of potential interest detected, *Aggregatibacter* spp*.*, *Cardiobacterium* spp., and *Eikenella corrodens*, which are usually part of a “healthy” microbiome, could be in rare cases associated with the development of infective endocarditis^42^. The presence of dental pathologies and the detection of amylases are perfectly in line with the consumption of a carbohydrate-rich, typically Neolithic, diet. Proteomics can also detect the immune response of the host, and several human proteins were observed including numerous components of the innate immune system, e.g., neutrophil degranulation and antimicrobial peptides (Supplementary Fig. S3). This provides confirmation at the molecular level of the fact that the individual had suffered from infections during his life^65–68^.

Due to the poor preservation of the remains during the archaeological investigation, the individual was initially identified as a female^4^. However, ancient DNA data and laboratory analysis of the skeletal remains led to an undoubted identification of the individual as a male, confirming the outcome of a recent anthropological investigation^6^. PCA projection of the genetic data on the dataset of ancient samples shows how SLB falls into the variability of the Italian and Mediterranean Neolithic, near the Peloponnesian and Anatolian Neolithics. This Neolithic genetic signature finds support on the uniparental haplogroups recovered from SLB: both K1a branches of mitochondrial DNA and G2a Y-chromosome sub-lineages have been indicated as part of the Neolithic genetic component that reached the European continent 8000 years ago, with G2a sub-haplogroups being the most widely distributed uniparental lineages among Neolithic Europeans^69,70^ (Fig. 4c). When compared to modern populations, SLB shifts in proximity of the Sardinian cluster, which has been reported to retain the greatest proportion of Neolithic ancestry among all Italian samples^71–74^. On the basis of modern data, the genetic prehistory of SI has been proposed to be the result of different migratory events from those that shaped the Eastern and Central Europe ancestrality, with Mediterranean genetic links between Aegean populations and SI that have been traced back to Neolithic period^75,76^. The genetic contributions that linked SI with the Greek islands and Anatolia have suggested the possibility that the Mediterranean could have served as a complementary crossroad for migratory events that occurred during the Neolithic^77^, even though such possibility cannot be fully explored with a single individual from Calabria. Nevertheless, the paleogenomic landscape of prehistoric SI is far from being understood, and new data are needed to better understand the legacy of ancient migrations in this region. The paleogenomic results presented in this work are certainly affected by the low number of SNPs used, but they represent another important step for archeomolecular studies in the Southern regions of the Italian peninsula.

The results of such an interdisciplinary study allowed us to reconstruct an almost complete bioarchaeological profile of the individual. Whether the funerary anomaly expressed in Grotta di Pietra Sant’Angelo could be related to necrophobic or ritual practices, such theories cannot be clarified through the outcome of this interdisciplinary research. However, some of the evidence we gathered suggests interesting possibilities. The absence of traces of trauma on skeletal remains and the presence of bacteria potentially related to inflammatory diseases, combined with the detection of human proteins of the innate immune system, could suggest the persistence of a non-specific inflammatory state with potentially fatal consequences. The possibility that the individual may have died away from his community would explain the unorthodox choice of burying the person in a site where no specific mortuary functions have been attested. Despite the lack of a clear funerary equipment, the burial itself could be defined as the expression of carefully planned social behaviour, especially considering the difficulties that arise in accessing the cavity. The contribution of geochemical methodologies such as stable isotopes analysis, not applied in this study, could be beneficial to better understand the provenance of the individual. Furthermore, the authors hope that in the near future new molecular data may be generated from Calabria, whose rich prehistory deserves to be explored through integrated approaches.

## Methods

All the experiments performed for the present work are in accordance with specific guidelines and regulations.

### Anthropology and paleopathology

The examination of the position of the bones was carried out in situ before the detachment of the skeleton from the ground following the methodological indications of Crubezy et al., 1990^78^ and Duday, 2006^28^. A preliminary anthropological investigation^6^ was focused on biological parameters assessment such as age-at-death, sex and stature; anthropological data, were acquired through macroscopic morphological observation and metric survey of the bones, according to the main methods reported in the literature^79–89^.

Paleopathological analysis of the skeleton was applied to identify and describe macroscopic abnormalities on bones and joints, both in the axial and appendicular skeleton (Supplementary Fig. S1). Paleopathological analysis was conducted by direct inspection, and medical computed tomography (acquisition parameters: 1.25 section thickness, reconstruction interval 0.6 mm, 120 kV and 100 mA) carried out at UO of Radiology at the Morgagni Pierantoni Hospital in Forlì. Visual assessments include identification of pathological lesions and their distribution within the affected bones and joints, and descriptive summary of the type of the abnormal features^49^. Descriptive analysis may provide important datasets for more in-depth paleopathological research.

### Microdebris analysis

Microdebris analysis was performed on dental calculus retrieved from the first right lower incisor, in which the presence of wear on the occlusal surface could indicate a use of the mouth as a "third hand". The protocol was based on previous studies^65,90^. Following the extraction of the calculus and a decontamination step to eliminate elements deriving from the burial soil, the sample was decalcified in a weak solution of hydrochloric acid (0.6 M HCl). This step dissolves the inorganic matrix and extracts all the microdebris that have been incorporated within the calculus throughout the life of the individual. The observation of the microdebris was carried out using a polarizing optical microscope with transmitted light. The slides were scanned at magnification of 160x, 400x, 1000 × using a Leica ICC50 W optical microscope. The microdebris (Supplementary Fig. S2) were divided into morphotypes based on the morphology and a name, a description and hypothesis on the origin and method of incorporation assigned to each. Any suggested identification is based upon their anatomical characteristics and comparison with modern reference material and key reference texts^90–99^.

### Protein analysis method

Sample preparation for paleoproteomics was carried out at the dedicated ancient protein laboratory (“Archaeobiomics”) at the University of Turin, following international guidelines for minimizing contamination and ensuring the authenticity of the sequences^100^. In brief, the sample (PALTO 247) was weighed (7.6 mg), washed in 200 μL ethylenediaminetetraacetic acid solution (EDTA, 0.5 M, pH = 8) for ten minutes, then demineralized in 500 μL 0.5 M EDT with gentle agitation (450 RPM) for eight days. The demineralized sample was processed following the SP3 protocol for low-protein samples^101^. Following the reduction of disulphide bonds with dithiothreitol (1 M aqueous solution) at 65 °C for 60 min and alkylation with iodoacetamide (0.5 M aqueous solution) at room temperature for 45 min, 30 μL of Sera-Mag SpeedBeads (1:1 mixture of hydrophobic and hydrophilic) were added to each of the extracts. To induce binding, 100% EtOH (HPLC-grade) was added to a final EtOH concentration of 50% and incubated at 24 °C for 5 min at ~ 1000 rpm. The tubes were then placed on a magnetic rack for separation, the supernatant removed and discarded. The proteins bound to the beads were cleaned with 80% EtOH (3X), exchanged to buffer (50 mM ammonium bicarbonate, pH 7.5–8) and the mixture sonicated for 30 s. After this step, trypsin was added (0.5 μg, Promega, proteomics grade) for overnight digestion at 37 °C and light shaking was applied (~ 1000 rpm). Afterwards, the extracts were centrifuged for 1 min, placed on a magnetic rack, the supernatants containing the digested peptides were transferred to separate tubes, acidified with 10% TFA (to a final TFA concentration of 0.1%) and the samples purified using C18 solid-phase extraction tips (Pierce, Thermo-Fisher). Eluted peptides were evaporated to dryness.

Liquid chromatography-tandem mass spectrometry (LC–MS/MS) analyses were performed at The Novo Nordisk Foundation Center for Protein Research, Faculty of Health and Medical Sciences, University of Copenhagen. The dental calculus sample (PALTO 247) was preceded by one lab control (ancient mollusk shell A) and two wash blanks and followed by one wash blank and one laboratory control (ancient mollusk shell control B). Dried peptide eluates were resuspended in 30 μL 0.1% formic acid in 80% acetonitrile (ACN) in water and transferred to a SpeedVac™ Concentrator (Thermo Fisher Scientific, Denmark) vacuum centrifuge at 40 °C until approximately 5 μL of the solution was left. 15 μL of 0.1% trifluoroacetic acid (TFA), 5% ACN was then added to each well. Samples were then separated on a 15 cm column (75 μm inner diameter) in-house laser pulled and packed with 1.9 μm C18 beads (Dr. Maisch, Germany) on an EASY-nLC 1200 (Proxeon, Odense, Denmark) connected to an Orbitrap Exploris 480 mass spectrometer (Thermo Scientific, Bremen, Germany) on a 77 min gradient. Four microliters of the sample/control were injected. LC and MS parameters were run according to the dental calculus method in Scorrano et al.^102^, with the HCD collision energy at 30%, RF Lens at 40%, and MS2 inject time of 54 ms to adjust for differences between the mass spectrometers.

Raw mass spectrometry data were searched against a combined database of Uniprot (Universal Protein Database, downloaded 06/12/2022) and eHOMD (expanded Human Oral Microbiome Database, downloaded 06/12/2022) protein sequences using the PEAKS Studio XPro software. Outputs of Spider searches carried out including all possible modifications and amino acid substitutions, were considered for further analysis. The thresholds for peptide and protein identification were set as follows: peptide score − 10lgP ≥ 30, protein score − 10lgP ≥ 40, de novo sequences scores (ALC%) ≥ 80, unique peptides ≥ 2. To ensure that all sources of contamination were considered, the results discussed here are based on the set of proteins which remain after all proteins found in the blanks and control are removed (the unfiltered results are reported in Supplementary Dataset).

The whole dataset was also used for estimating the extent of deamidation, both overall and site specific, using the DeamiDATE tool^103^. The raw MS data were first searched using MaxQuant v.1.6.3.4^104^ with a database made of all positive protein hits from the PEAKS search. Carbamidomethyl (C) was set as a fixed modification; oxidation (M), deamidation (NQ), pyroglutamic acid (QE), and hydroxyproline were all set as variable modifications. Digestion was set to trypsin with a maximum of two missed cleavages. The false discovery rate (FDR) was set to 1% and the minimum score cut-off for modified and unmodified peptides was 50. All other parameters were left for the default for orbitrap mass spectrometers. All laboratory and handling contaminants were removed from the results.

### Ancient DNA analysis

The analyses were conducted at the Ancient DNA Laboratory of the Department of Cultural Heritage (University of Bologna), following strict standards for paleogenomic workflow^105,106^. A second lower left molar was isolated for ancient DNA extraction (SLB2). The tooth was smoothly cleaned with 4% HCl, rinsed in 80% EtOH and then sterilized under UV-light for 20’. 1–2 mm external surface of the root was then abraded with a rotatory blade to remove superficial layer, and 60 mg of cementum and dentin powder was collected by drilling the root at low speed with dental bit. For metagenomic analysis, a 15 mg fragment of dental calculus (SLBT) was sampled from the second lower left incisor and after 20’ UV irradiations, it was powdered on sterilized mortar for DNA extraction. DNA isolation was performed as in Dabney et al. (2013) with slight laboratorial modifications as in Cilli et al. (2020)^107^ and molecular concentrations were measured on QuBit fluorometer. Blank controls were processed along with the samples during every phase of the analysis. Single stranded libraries were then built for both extracts^108^, pooled at equimolar amounts with other samples and screened for endogenous DNA on HiSeqX Ten 2 × 150 bp lane. Once confirmed the presence of authentic ancient DNA, SLB sample was sequenced a second time on HiSeqX Ten 2 × 150 bp lane, generating > 500 million reads.

Raw data were processed with Paleomix^109^: the fastq files were filtered with AdapterRemoval^110^ that removed adapters and reads shorter than 30 bp. Reads were then separately aligned to the Human Reference Genome (GRCh37) and to an elongated version of the rCRS using Circular Mapper v1.93.5 (https://github.com/apeltzer/CircularMapper) and bwa v0.7.17 aln algorithm^111^, with minimum mapping quality ≥ 20, seeds disabled and parameter − n set to 0.01. PCR duplicates were automatically removed and the authenticity of the reads was confirmed by evaluating the patterns of deamination at both ends of the reads with mapDamage v2.2.1^112^. Modern human contamination was estimated at mitochondrial and X-chromosome level via Schmutzi^113^ and ANGSD v0.939^114^ respectively. We used Schmutzi to generate the consensus sequence of the mitochondrial DNA, and the haplogroup was assigned with HaploGrep2^115^ and Haplofind^116^. The—rescale option from mapDamage was used to downscale the quality values of likely-damaged positions of the reads due to ancient DNA damages. Then, a list of informative SNPs for Y-chromosome was downloaded from the International Society of Genetic Geneaology (https://isogg.org/tree/), and the software yhaplo (https://github.com/23andMe/yhaplo) was used to identify Y-chromosome haplogroup from reads with mapping quality scores ≥ 30 and bases with quality scores ≥ 30. Biological sex of the individual was estimated by computing the ratio of reads aligning to the X-chromosome as a portion of the total sequences mapping to the autosomes using the R script provided in Mittnik et al. 2016^117^. The results were also compared with the sex assignment approach provided by Skoglund et al. 2013^118^, that calculates the ratio of reads mapping to Y-chromosome as a portion of the alignments to the sexual chromosomes. A maximum likelihood phylogenetic tree was reconstructed with the software MEGA X^119^ using a dataset of 130 Chalcolithic to Middle Late Neolithic Eurasians of haplogroup K (Supplementary Table S5), by setting the substitution model HKY + I + G, assessed by JModelTest2^120^ and running 1000 bootstrap repetitions. The Median Joining Network (Supplementary Fig. S5) was performed with Network software v.10 (www.fluxus-engineering.com) using default parameters on a set of 53 K1a (n = 19), K1a + 150 (n = 3), K1a + 195 (n = 30) and K1f. (n = 1) individuals (Supplementary Table S6). Poly-C stretches, AC-indels at positions 303–315, 515–524, 16,180–16,193 and hypervariable position 16,519 were excluded from the phylogenetic reconstruction.

Pseudohaploid genotypes were called from reads with mapping quality ≥ 30 using PileupCaller, (https://github.com/stschiff/sequenceTools) that randomly selects one high quality base (phred base quality score ≥ 30) based on the 1240 K reference panel. Minimum base and mapping quality of 30 filters were applied for the samtools mpileup command, and pseudo-haploid data generated by randomly calling one allele from each covered site using the pileupCaller script. For the contextualization of SLB in the ancient Mediterranean variability, a dataset of 905 ancient individuals was selected from the 1240 K—Human Origins SNP Array (https://reich.hms.harvard.edu/allen-ancient-dna-resource-aadr-downloadable-genotypes-present-day-and-ancient-dna-data). For each of the Mediterranean populations observed in the original dataset, only individuals for a period between the Mesolithic and Bronze Age were selected, excluding all those individuals that were referred to as duplicate, related, contaminated, and low-covered.

A representative panel of 1257 modern individuals to be used as a reference base for the PCA projection was extracted from the HumanOrigins dataset. These contain a lower number of markers (~ 600 K SNPs) but have the advantage of having a good coverage of all populations and of being already merged with the ancients, which simplifies the procedure and avoids further reductions in the number of variants when merging. Therefore, we obtained a comparison panel of 2162 IND and 431,156 SNPs (Supplementary Table S7) from ancient and modern Euro-Mediterranean populations. The data of ancient SLB were then merged with the custom dataset, for a total 92,322 SNPs remaining after further pruning (using a 200 25 0.4 filter). Finally, we did the PCA projection of the ancient individuals with SLB on the modern Euro Mediterranean HOs, and the resulting plots were divided in four temporal screens, namely Mesolithic, Neolithic, Chalcolithic and Bronze Age (Supplementary Fig. S6).

### Modern local samples genotyping and data curation

Genotyping of 20 modern inhabitants of the nearby municipality of San Lorenzo Bellizzi was included in this study. All these samples were selected according to the grandparents and founder surname criteria, to guarantee at least three generations of ancestry in the area, and by excluding related individuals. All the procedures concerning the modern samples were approved by the Bioethics Committee of the University of Bologna on 08/04/2013. All donors provided a written informed consent to data treatment and project objectives. The study was designed and conducted in agreement with the ethical principles for research involving human subjects stated by the WMA Declaration of Helsinki. Saliva samples were collected with the Oragene-DNA Self Collection Kit OG500 (DNA Genotek Inc., Ottawa, Ontario, Canada). Genomic DNA was extracted following manufacturer’s recommendations and quantified with dsDNA BR Assay Kit and Qubit fluorometer (Life Technologies, Carlsbad, CA).

DNA samples were then genotyped for the 713,014 SNPs included in the HumanOmniExpress BeadChip (Illumina, San Diego, CA, USA), in the Center for Biomedical Research & Technologies of the Italian Auxologic Institute (Milan, Italy). The 20 samples were merged with 737 already published individuals, representative of the genetic variability along the Italian peninsula, as well as Sardinia^121^. Merging was performed with the PLINK v.1.9^122^ by taking into consideration only autosomal SNPs that were shared across the two datasets, obtaining a new set of 757 individuals and 254,709 variants. A standard quality control was then carried out with functions implemented in the same PLINK package to ensure a high quality for the resulting final dataset of modern individuals. Specifically, data missingness was checked across SNPs and across individuals with a threshold of 0.05, leaving 254,614 SNPs and 757 individuals; the respect of Hardy–Weinberg equilibrium was also inspected, imposing a Bonferroni correction for multiple testing to the standard threshold of 0.01, so that the new threshold is equal to 3.9275e−8. Variants in linkage disequilibrium were also tested using a sliding window approach with a window of size 500 SNPs, a step of 50 SNPs and a LD threshold of 0.1 and, if detected, one variant was randomly removed from the dataset. The complete QC returned 49,586 SNPs and 757 individuals. Finally, the dataset (757 + SLB individual) was projected on the modern genetic background using SmartPCA software from the EIGENSOFT package using lsqproject: YES option.

### Microbiome analysis

We applied HOPS^31^ for characterizing the metagenomic profile of the shotgun reads. The sequences were first aligned to the database using a modified version of MALT^123,124^. In particular, the database was created by malt-build using the “representative” and “reference” bacterial (n = 11,270) and archaeal (n = 408) genomes downloaded from NCBI RefSeq on November 16, 2020. The reads assigned to bacterial genomes were extracted with the MaltExtract tool and realigned to their respective reference genomes to evaluate edit distances, coverage distributions, and post-mortem DNA damage patterns^31,125,126^. To this aim, we used MapDamage^112^ to estimate deamination rates. Post-mortem degradation score (PMDS) distributions were computed using PMDtools^127^. The breadth and depth of coverage were estimated using bedtools^128^. Edit distances for all sequences compared to their references were used for calculating the negative difference proportion score (−Δ %) as previously described by Hübler and colleagues (2019)^31^. A − Δ % > 0.9 was considered as relevant for a declining distribution related to ancient DNA profile. Taxa with more than 300 assigned reads, more than 50 reads with PMDS > 1, − Δ % > 0.9 and showing patterns of C-T transition at 5′ were considered to be of ancient origin. The MALT reference database was built using the malt-build command and selecting the “representative” and “reference” bacterial (n = 11,270) and archaeal (n = 408) genomes from NCBI RefSeq (November 16, 2020). The same pipeline were used on a dataset of 114 dental calculi derived from previous studies^35,36^, including Neanderthal, and human from different era (pre-agricultural, neolithic, pre-antibiotic period and modern day humans). Sequencing data have been downloaded from the ENA repository (https://www.ebi.ac.uk/ena/browser/home) under project accession IDs PRJEB34569 and PRJEB44313. Raw reads from the respective repositories were downloaded and the HOPS pipeline applied using the same parameters listed above. The resulting bacterial counts normalized for sequencing depth were used to perform a PCoA analysis with the Bray–Curtis distance.

Screening of potential bacterial pathogens were also performed, by searching for specific traces of their DNA within the results of the same HOPs pipeline describe above. In particular, we seek for genomic traces of pathogens claimed by the National Institute of Allergy and Infectious Diseases (NIAID) as most relevant infectious diseases for human health^129^. We selected those bacteria, whose genomes were included within the HOPS database: *Bacillus anthracis, Bacillus cereus, Borrelia, Brucella, Campylobacter jejuni, Clostridioides difficile, Clostridium botulinum, Clostridium perfringens, Clostridium tetani, Corynebacterium diphtheriae, Escherichia coli, Francisella tularensis, Haemophilus, Klebsiella pneumoniae, Legionella, Listeria, Mycobacterium haemophylum, Mycobacterium leprae, Mycobacterium tuberculosis, Mycoplasma, Neisseria gonorrhoeae, Neisseria meningitidis, Porphyromonas gingivalis, Pseudomonas aeruginosa, Salmonella, Serratia marcescens, Shigella, Staphylococcus aureus, Streptococcus gordonii, Streptocossus mutans, Streptococcus pneumoniae, Treponema pallidum, Vibrio cholerae, Yersinia enterocolitica* and *Yersinia pestis*. Taxa with more than 10 CPM (reads matching to the specific reference per million of sequenced reads) were subjected to the same analysis reported above (negative proportion score, coverage distributions, and post-mortem DNA damage patterns^31,125,126^), for validating their ancient origin.

## Supplementary Information

Supplementary Information 1.

Supplementary Information 2.

Supplementary Tables.

## Data Availability

All data produced or analyzed during the current study are available from the corresponding authors on reasonable request. Raw genetic data from tooth and calculus are deposited at the European Nucleotide Archive under project accession number PRJEB58818. The reconstructed mitochondrial genome from SLB has been made available in GenBank under accession number OQ301533. The datasets of ancient proteins detected from dental calculus have been deposited to the ProteomeXchange Consortium via the Proteomics Identifications Database (PRIDE) partner repository with the identifier PXD039654 [Username: reviewer_pxd039654@ebi.ac.uk; Password: iFHQ4lrk]. The skeletal remains are stored at the Speleo-archaeological Research Center of San Lorenzo Bellizzi (CS).
