# Supplementary material for: Bioarchaeological and paleogenomic profiling of the unusual Neolithic burial from Grotta di Pietra Sant’Angelo (Calabria, Italy)
Source: Sci Rep. 2023 Jul 24;13:11978. doi: 10.1038/s41598-023-39250-y (PMC10366206; doi:10.1038/s41598-023-39250-y)
Supplement: Supplementary file 2 — Supplementary Information 2. [file 41598_2023_39250_MOESM2_ESM.pdf]

Supplementary Information

Bioarchaeological and paleogenomic profiling of the unusual Neolithic burial from Grotta di Pietra Sant'Angelo (Calabria, Italy)

Francesco Fontani (1), Rosa Boano (2), Alessandra Cinti (2), Beatrice Demarchi (2), Sarah Sandron (2), Simone Rampelli (3), Marco Candela (3), Mirko Traversari (1), Paolo Abondio (1), Adriana Latorre (1), Rocco Iacovera (1), Stefania Sarno (4), Meaghan Mackie (5), Matthew Collins (5), Anita Radini (6), Chantal Milani (7), Enrico Petrella (8), Emanuela Giampalma (8) Antonella Minelli (9), Felice Larocca (10,11), Elisabetta Cilli (1) and Donata Luiselli (1)

1. Department of Cultural Heritage, University of Bologna, via degli Ariani 1, 48121 Ravenna, Italy
2. Department of Life Sciences and Systems Biology, University of Turin, via Accademia Albertina 13, 10123 Torino, Italy
3. Department of Pharmacy and Biotechnology, University of Bologna, Via Belmeloro 6, 40126 Bologna, Italy
4. Department of Biological, Geological and Environmental Sciences, University of Bologna, via Selmi 3, 40126 Bologna, Italy
5. The Novo Nordisk Foundation Center for Protein Research, Faculty of Health and Medical Sciences, University of Copenhagen, Blegdamsvej 3B, 2200 København, Denmark
6. The Globe Institute, Faculty of Health and Medical Sciences, University of Copenhagen, Øster Farimagsgade 5, 1353 København, Denmark
7. School of Archeology, University College Dublin, Belfield, Dublin 4, Ireland
8. McDonald Institute for Archaeological Research, University of Cambridge, Downing Street, CB2 3ER Cambridge, United Kingdom
9. SIOF – Italian Society of Forensic Odontology, Strada degli Schiocchi 12, 41124 Modena, Italy
10. Radiology Unit, Morgagni-Pierantoni Hospital, AUSL Romagna, via Carlo Forlanini 34, 47121 Forlì, Italy
11. Department of Humanities, Education and Social Sciences, University of Molise, via Francesco De Sanctis, 86100 Campobasso, Italy
12. Speleo-Archaeological Research Group, University of Bari, Piazza Umberto I 1, 70121 Bari, Italy
13. Speleo-Archaeological Research Centre "Enzo dei Medici", Via Lucania 3, 87070 Roseto Capo Spulico (CS), Italy

Supplementary Information

Supplementary Information on microscopy analysis

Supplementary Figures

Supplementary Information on Microscopy Analysis

The analysis by light microscopy of microdebris from dental calculus yielded microremains of low diagnostic nature, some of which could not be assigned with certainty to a Kingdom, therefore are described below as un-diagnostic.

Diagnostic debris from the Kingdom Plantae

Microdebris attributed to the Plant Kingdom consisted of:

- poorly-preserved starch granules, which could not be assigned to a specific Family or Tribe. Such debris was identified as starch due to the overall aspect under the microscope, size, presence of hilum and due to the observation of residual birefringence (see Fig. A). Some debris could also be potentially starch, but lacked birefringence. The poor status of preservation could be due to the processing and cooking of starchy food (Henry et al., 2009).
- a small fragment of plant tissue was also found, again lacking any diagnostic features to allow for secure identification (Fig. B). Such remains could potentially be the result of the consumption of leafy crops.
- a plant trichome (i.e. a plant hair), common on leaves of many plants (Fig. C).
- Two fibers, one elongated and hyaline in aspect while the other showed features similar to those sometimes found in some bast fibers, but the preservation of the fiber itself makes the identification doubtful (Figs. D and E).

Un-identified and un-diagnostic remains

A few particles of microdebris were retrieved from dental calculus, which could not be identified either for their status of preservation or because they were so small that they lacked any diagnostic features to support their identification. Among them there were:

- microdebris of mineral origin, often described in literature as mineral grit (Fig. F).
- some dark brown to black, sub-round and sub-angular particles, thought to be 'burnt debris' (Figure G). These particles had no feature visible by light microscopy that could help assign them to the category of micro-charcoal, however they are most likely the result of the burning of organic matter.

The above microdebris has been recorded and as reference collections improve all the time, it cannot be excluded that it will be possible to identify them in the future.

Supplementary Figures

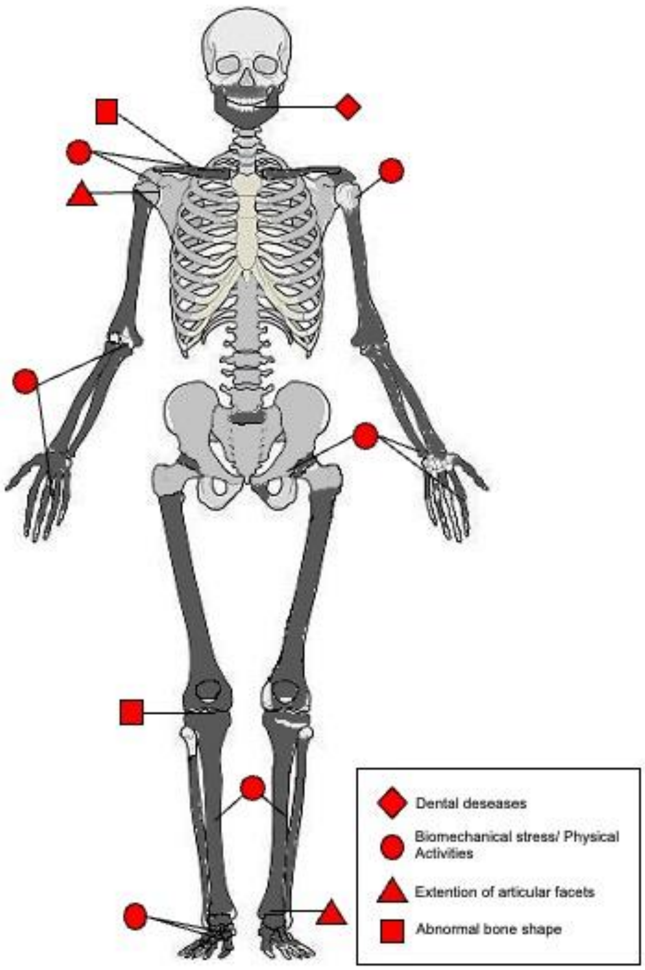

Supplementary Figure S1: Macroscopic abnormalities found in bones and joints of the axial and appendicular skeleton of the individual from Grotta di Pietra Sant'Angelo.

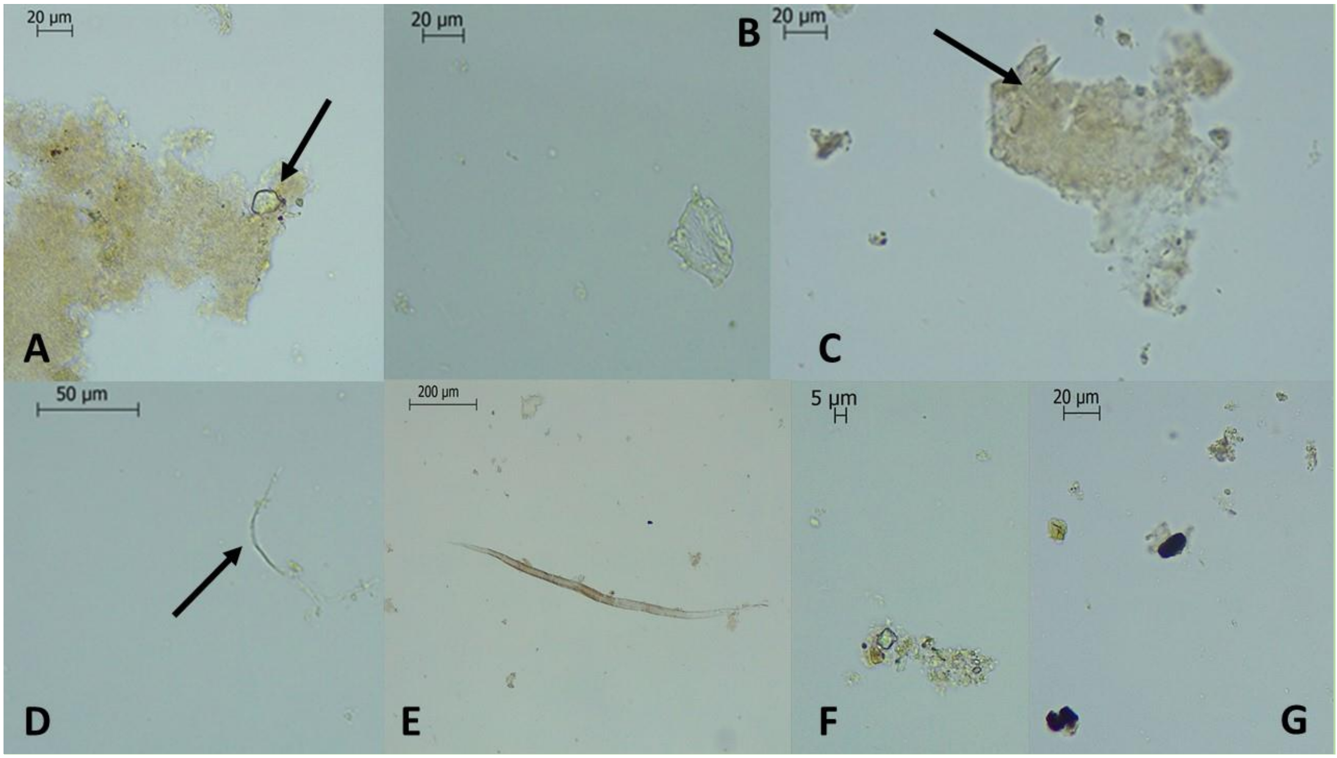

Supplementary Figure S2: Example of starch granule, found in situ in the dental calculus fleck. B) Small fragment of plant tissue. C) Trichome (plant hair). D) and E) fibers. F) mineral grit. G) Burnt debris - note the lack of anatomical features necessary to assign the microdebris securely to microcharcoal. Pictures by Anita Radini.

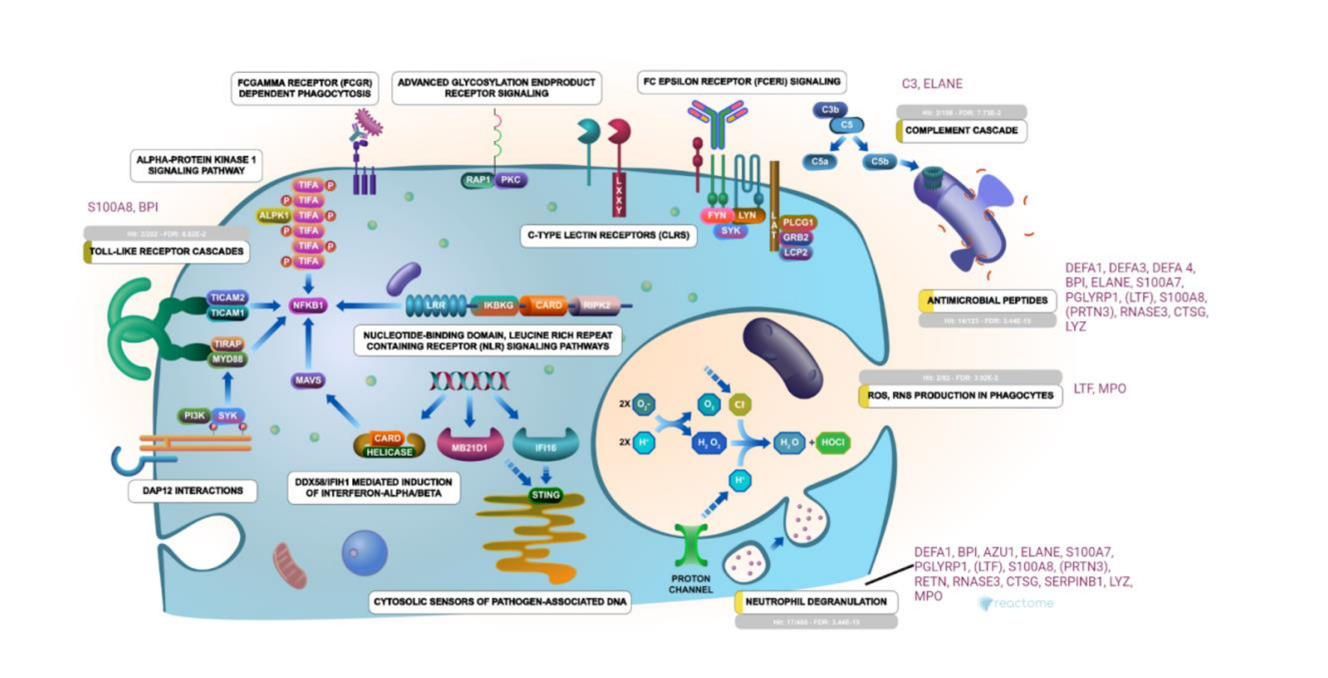

Supplementary Figure S3: The innate immune system reactome for *Homo sapiens*; gene names corresponding to proteins detected in the ancient oral microbiome of SLB are shown in red. Gene names in parenthesis refer to proteins which yielded lower-than-expected deamidation levels. Figure created by Beatrice Demarchi on Reactome and modified in biorender.com.

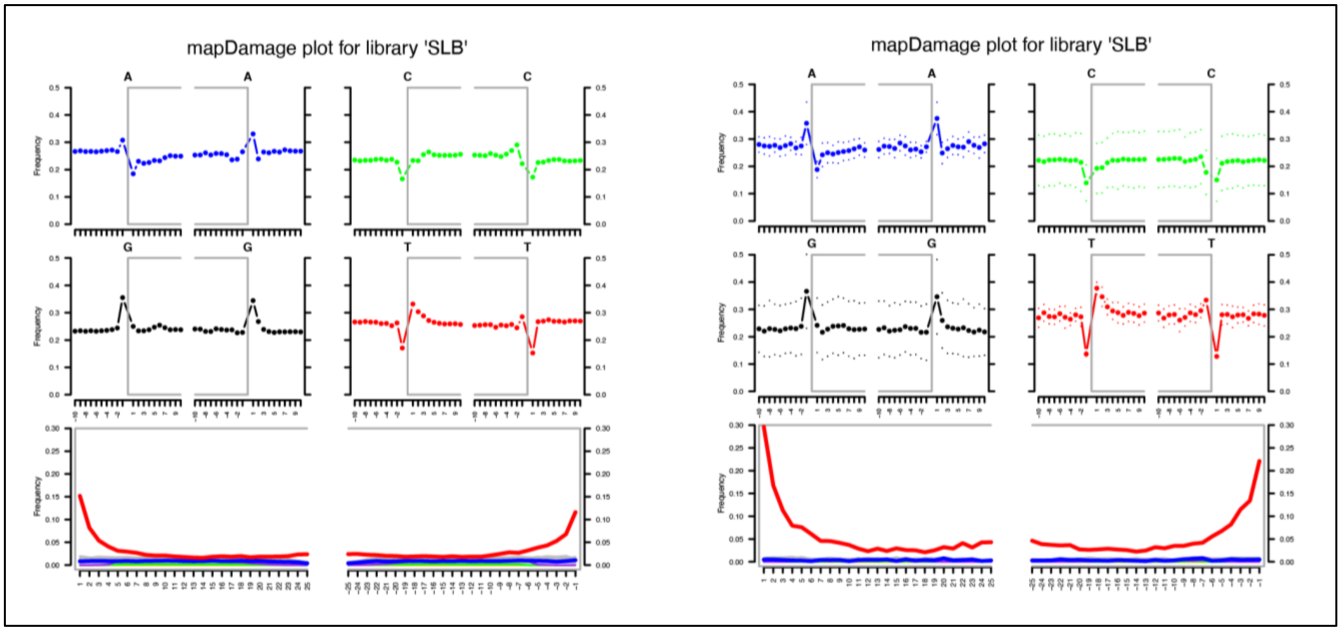

Supplementary Figure S4: Misincorporation plots produced by mapDamage from whole-genome (left) and mitochondrial genome ≥q30 mapped reads (right).

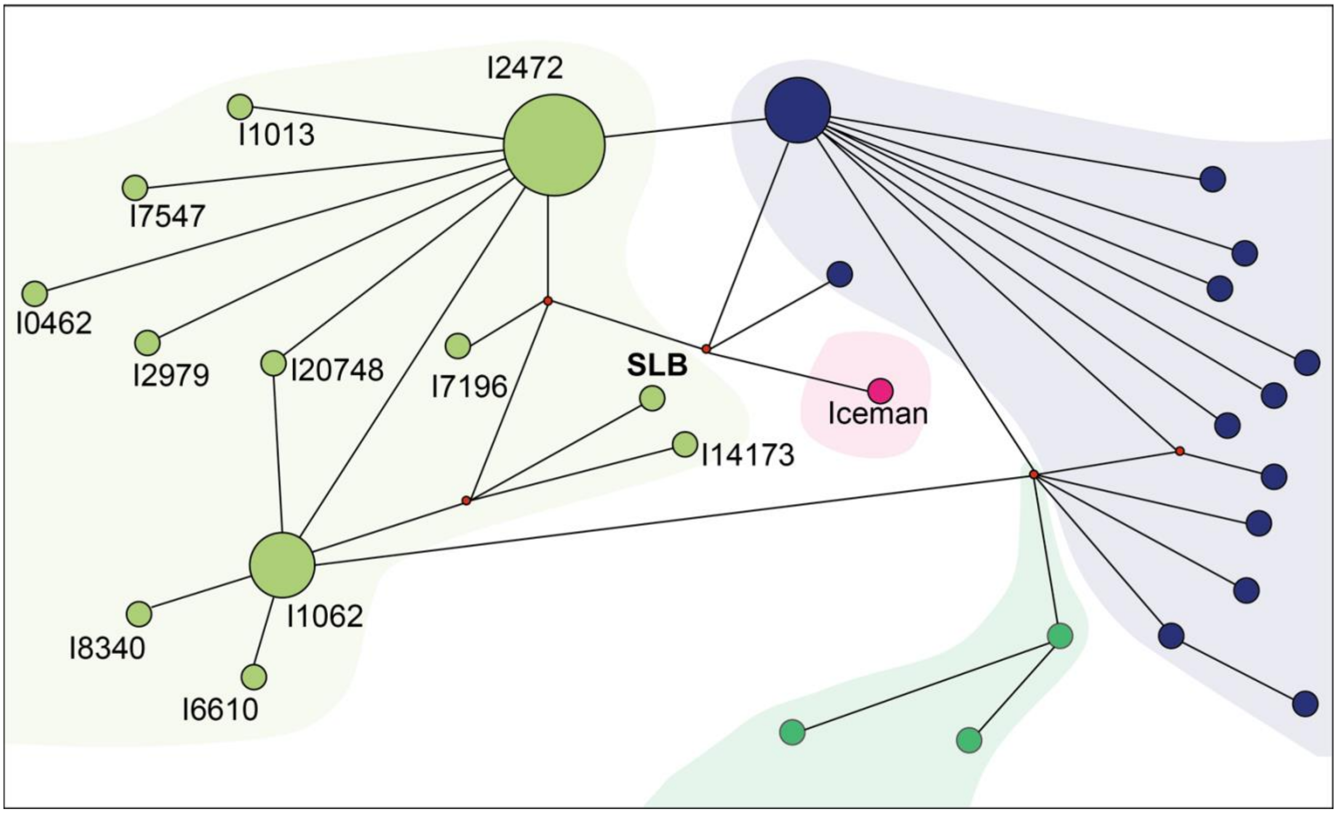

Supplementary Figure S5: MJN performed on a dataset of K1-related prehistoric individuals (K1a in blue; K1+195 in olive green; K1a+150 in bright green) confirms the attribution of SLB to the K1a+195 mitochondrial haplogroup, with the individual being few nodes far from the Iceman, whose rare mitochondrial haplogroup has been identified only within the Alps area (K1f, pink dot).

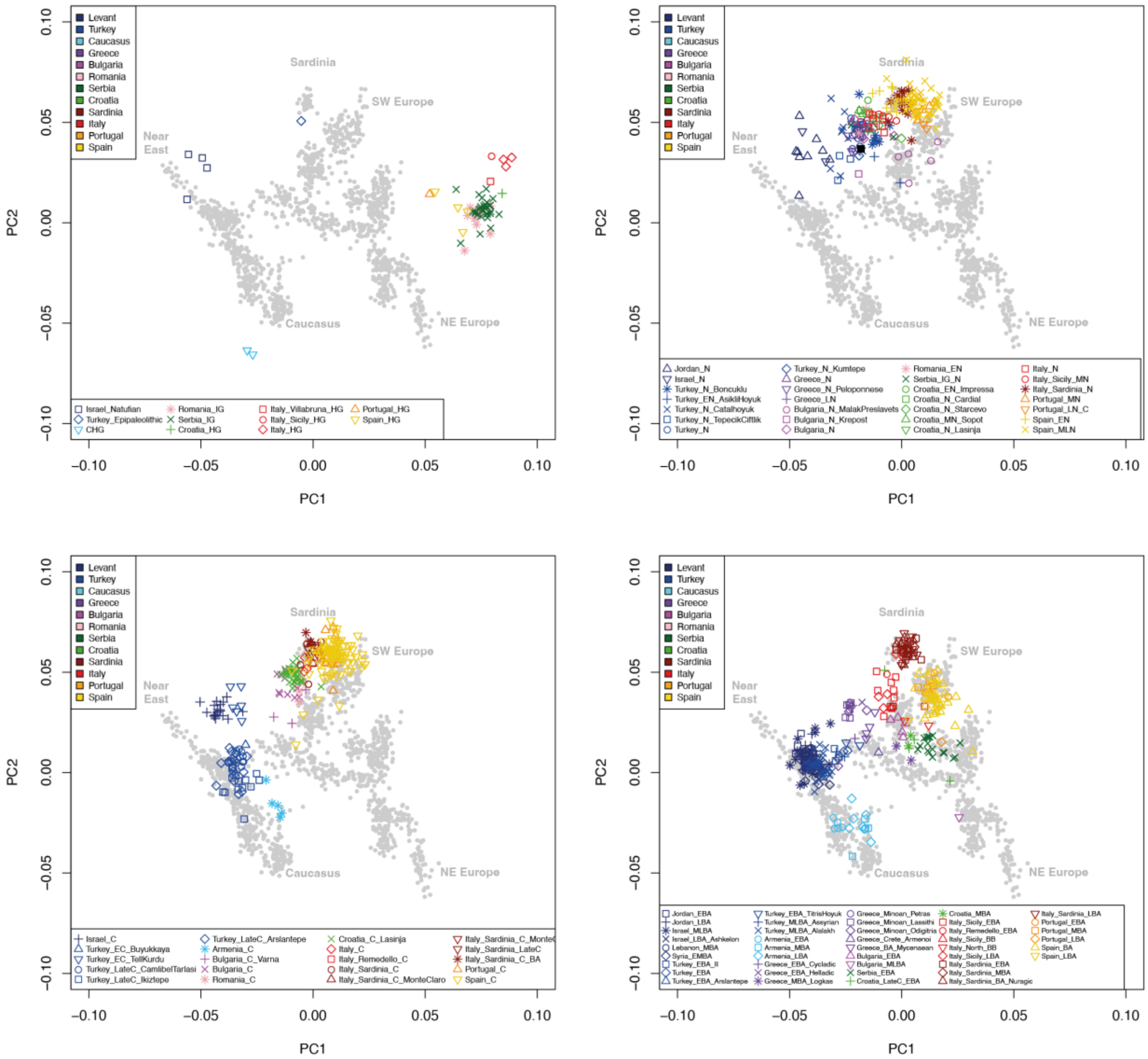

Supplementary Figure S6: PCA projections of the ancient individuals on the modern Euro-Mediterranean HO dataset (grey dots). The resulting plots are divided in four temporal screens: Mesolithic (top left), Neolithic (top right) with SLB (black square), Chalcolithic (bottom left) and Bronze Age (bottom right).
